# Aquaporins in Cereals—Important Players in Maintaining Cell Homeostasis under Abiotic Stress

**DOI:** 10.3390/genes12040477

**Published:** 2021-03-25

**Authors:** Marzena Małgorzata Kurowska

**Affiliations:** Institute of Biology, Biotechnology and Environmental Protection, Faculty of Natural Sciences, University of Silesia in Katowice, Jagiellońska 28, 40-032 Katowice, Poland; marzena.kurowska@us.edu.pl

**Keywords:** aquaporins, cereals, abiotic stress, drought, salinity, cold, gene expression study, genetic modification, plants

## Abstract

Cereal productivity is reduced by environmental stresses such as drought, heat, elevated CO_2_, salinity, metal toxicity and cold. Sometimes, plants are exposed to multiple stresses simultaneously. Plants must be able to make a rapid and adequate response to these environmental stimuli in order to restore their growing ability. The latest research has shown that aquaporins are important players in maintaining cell homeostasis under abiotic stress. Aquaporins are membrane intrinsic proteins (MIP) that form pores in the cellular membranes, which facilitate the movement of water and many other molecules such as ammonia, urea, CO_2_, micronutrients (silicon and boron), glycerol and reactive oxygen species (hydrogen peroxide) across the cell and intercellular compartments. The present review primarily focuses on the diversity of aquaporins in cereal species, their cellular and subcellular localisation, their expression and their functioning under abiotic stresses. Lastly, this review discusses the potential use of mutants and plants that overexpress the aquaporin-encoding genes to improve their tolerance to abiotic stress.

## 1. Introduction

**Figure 1:**
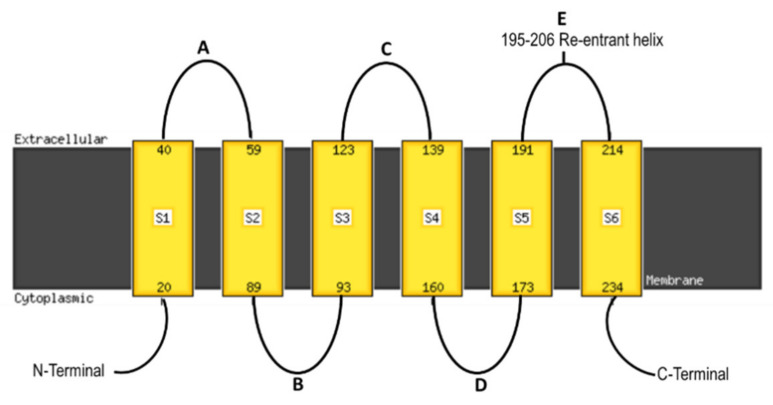
Transmembrane helix prediction for the HvTIP2;3 protein sequence of barley (HORVU7Hr1G081770) that was obtained using Phyre2 software [14]. S1–S6: six transmembrane helices, A–E: five connecting inter-helical loops.

Aquaporins (AQP), which are members of the major intrinsic proteins (MIP) superfamily, facilitate the bi-directional flux of water and non-aqua substrates across the cell membranes [1,2,3,4]. The history of studies on AQPs is quite interesting, and two groups were involved in the breakthrough research: Agre’s and Benga’s group. The first water channel protein that was discovered and characterised from human erythrocytes was CHannel-forming Integral membrane Protein of 28 kDa (CHIP28), which was later renamed Aquaporin-1 (AQP1) after its water conductivity was shown by expression in *Xenopus* oocytes [5]. The Nobel Prize in Chemistry for 2003 was awarded to two scientists, Peter Agre and Roderick MacKinnon, who had made fundamental discoveries concerning the channels in the cell membranes. Additionally, the water channel protein (WCP) had also been identified in situ in the human red blood cell (RBC) membrane earlier by Benga’s group [6,7]. In turn, the first AQP in plants was identified in soybeans—GmNOD26 [8], while the ability to conduct water in plants was demonstrated in Arabidopsis for AtTIP1;1 (gamma-TIP) by its heterologous expression in the *Xenopus* oocytes for the first time [9]. These transmembrane proteins form pores in the lipid bilayers of archaea, bacteria, fungi, plants, non-mammalian metazoans and mammalians including humans. Although it has been suggested that AQPs are multifunctional proteins, their structure is unique across all of the kingdoms of living organisms. The molecular weight of the members of the AQP family ranges from 23 to 31 kDa [10]. Despite some sequence diversity, all MIPs share a similar three-dimensional structure that consists of six transmembrane helices (S1 to S6) with five connecting inter-helical loops (A to E) and two half helices that contain one highly conserved Asn-Pro-Ala (NPA) motif each, which forms a functional pore (Figure 1). The hydrophobic NPA motif is localised at the first intracellular (loop B) and the third extracellular loops (loop E) [10]. The NPA motif and the aromatic/arginine selectivity filter (Ar/R SF) that includes four amino acids (aa) towards the extracellular side form two narrow regions within a channel. These two constricted regions are very important to the transport selectivity of the channels, which were predicted based on structural knowledge combined with simulation studies [11,12]. The NPA motif not only plays a role in regulating membrane transport, but also in protein localisation [13].

**Figure 2:**
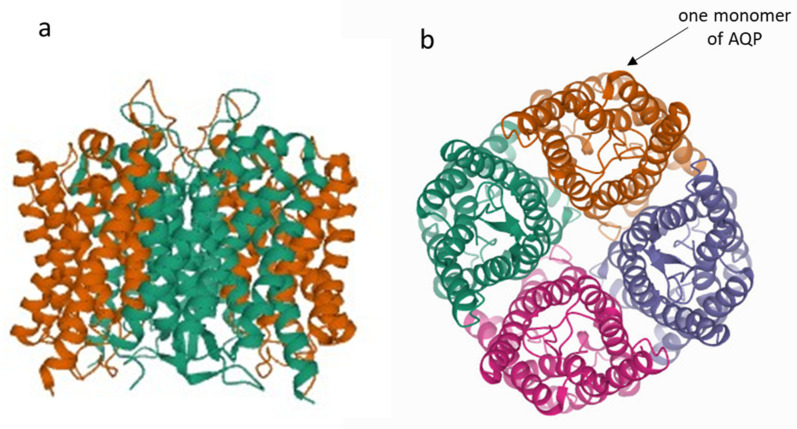
The structure of aquaporin represented by the crystal structure of Arabidopsis AtPIP2;4 (PDB 6QIM) [20]. The view from different sides of the holoprotein shows that it consists of monomers. (**a**) A side view of an asymmetric unit. (**b**) Tetrameric assembly from the cytoplasmic side.

All of the MIP sequences take on a typical hour-glass MIP helical fold with six transmembrane helices and two half-helices [15]. This fold was maintained during evolution with the conservation of around 40 positions within the transmembrane region [15]. The stability of the functionally important half-helix is modulated by stabilising the intra-helical salt-bridge interaction and/or two helix-destabilising residues glycine and proline, which was demonstrated by analysing the loop E region of 1468 MIP sequences and their structural models from six different groups of organisms [16]. In the cell membrane, the MIPs are grouped as homotetramers that are located in the lipid bilayer (Figure 2). Each monomer functions independently as a single channel pore [17]. In plants, the crystal structure of only two MIPs is available in the Protein Data Bank [18]; the spinach (*Spinacia oleracea*) aquaporin SoPIP2;1 was in its closed confirmation at a 2.1 Å resolution and its open confirmation at a 3.9 Å resolution [19], and the Arabidopsis aquaporins AtPIP2;4 at a 3.7 Å resolution [20]. All of these were determined using X-ray diffraction and exhibited a structural identity. When the crystal structure of a protein is not available, it is possible to use the bioinformatic tools such as Phyre2 to predict and analyse the protein structures, and then use MOLES 2.5 to quickly locate and characterise the pores [21].

The functions and regulation of the majority of MIP channels have not yet been fully characterised [15]. Some AQP not only facilitate the transport of water but also non-aqua substrates such as ammonia, antimony, arsenite, carbon dioxide (CO_2_), formamide, glycerol, hydrogen peroxide (H_2_O_2_), lactic acid, micronutrients (silicon and boron), oxygen (O_2_) and urea [10,22]. In plants, AQPs are present in almost all organs including the roots, leaves, stems, flowers, fruits, seeds, dry seeds [2,23,24], pollen [25,26,27,28], anther and specific cells such as guard cells [29,30].

In field conditions, crops are challenged by a wide range of abiotic stresses. Environmental stresses such as drought, adverse temperatures (cold or heat) and salinity are major causes of yield losses in crops worldwide [31,32,33,34,35]. The impact on yields results in a decrease in the harvest index, a shortened life cycle of crops and changes in the seed number, size and composition. Furthermore, these effects are more severe when the stress occurs during the reproductive stage of a plant [35]. In nature, different stresses often occur together, which further exacerbates their effect. Episodes of prolonged drought coupled with heat waves is one of the possible stress combinations that affects crop yield [35]. The response of plants is quite complicated, and many mechanisms are initiated simultaneously in order to restore cellular homeostasis and promote survival [36]. All of the main abiotic stresses such as drought, cold, heat and salinity cause an imbalance in the water status in cells, tissues and whole plants. Regulating the flow of water through the membranes is one of the main mechanisms via which cells can maintain their homeostasis under stress conditions and water channels that are called aquaporins (AQP) are involved in this process [37].

The role of aquaporin in physiological processes, stress tolerance and their regulation in plants has been extensively and recently reviewed [38,39,40,41,42,43,44,45,46]. The aim of this manuscript is to focus on the aquaporins only in cereal species, their diversity, expression pattern and roles under abiotic stress. Cereals are important plants in agriculture, which might be used as human food, animal feed and in the brewing industry. The potential use of mutants and plants that overexpress the aquaporin-encoding genes to improve cereals tolerance to abiotic stress is also discussed.

## 2. Diversity of Aquaporins in Cereal Species

A large number of *MIP* genes have been identified in the genomes of different plant species. In plants, the MIP family contains around 2008 members that are deposited in the MIPDB (Major Intrinsic Proteins Database) [47], and their sequences range from 35 to 854 aa. Depending on the membrane localisation and amino acid sequence, the MIPs in higher plants including AQPs are typically divided into five subfamilies: the plasma membrane intrinsic proteins (PIP), tonoplast intrinsic proteins (TIP), nodulin-26-like proteins (NIP), small, basic intrinsic proteins (SIP) and the uncategorised X intrinsic proteins (XIP) [10]. However, in the monocot plant species, the XIP subfamily was lost during the evolution [41]. The PIPs, NIPs and XIPs are mainly localised in the plasma membrane, while the TIPs are localised in the tonoplast—a membrane of the vacuole. The SIPs and some NIPs have been found localising to the endoplasmic reticulum [22,28,48]. Some TIPs and SIPs have been predicted to be localised to the inner envelope and thylakoids [22]. Such a location was detected for TIP1;1, TIP1;2 and TIP2;1 in Arabidopsis [49,50,51]. In cereals, the number of MIP sequences that have been identified in genomes ranges from 33 to 113 in rice and wheat, respectively (Table 1). In other plant species, for example in Arabidopsis (*Arabidopsis thaliana*), cotton (*Gossypium arboretum*), cucumber (*Cucumis sativus* L.), olive trees (*Olea europaea* var. *sylvestris* and cv. *Picual*), potato (*Solanum tuberosum* L.), tabacco (*Nicotiana tabacum*) and tomato (*Solanum lycopersicum* L.), a large number of *AQP* genes have also been identified in genome 38, 53, 39, 52, 79, 41 and 47, respectively [52,53,54,55,56,57,58]. Throughout evolution, plants have kept a high number of AQPs and regulation mechanisms, and it might be an advantage when facing developmental and environmental challenges [59].

## 3. Study of the Expression and Role of Aquaporins in Maintaining the Cell Homeostasis of Cereals under Abiotic Stress

Plants are immobile; consequently, a quick and adequate change of the physiological processes in response to changes in environmental conditions is essential for their growth and productivity [68]. In plants, AQPs mediate water transport in both the roots and shoots, which is associated with the impact on plant hydraulics, transpiration and soil water conservation [67]. The function of aquaporins during the growth of plants and their abiotic stress responses can generally be considered as being dependent on (a) the developmental stage; (b) the organ: roots, leaves, stems, flowers, fruits, seeds, dry seeds, pollen, anther and specific cells such as the guard cells; (c) their subcellular localisation: plasma, tonoplast, endomembrane, peribacteroid membrane or (d) the transporting substrate: water, glycerol, CO_2_, urea, ammonia, H_2_O_2_, boron, silicon, arsenite, antimonite, lactic acid or O_2_ [10]. The variety of substrates that are transported by AQPs reflects the variety of physiological processes that are regulated by these proteins. Photosynthesis in plants is regulated by AQPs through their effect on CO_2_ transport in the membranes of the chloroplast and thylakoid [69,70,71]. Another example is that by facilitating the urea and NH_4_^+^/NH_3_ uptake, the AQPs ensure the N homeostasis [71,72]. The enabling of H_2_O_2_ diffusion by AQPs seems to be very important during the stress response when the reactive oxygen species are produced, while on the other hand the transport of this signalling molecule could have important functions in plant development and the regulation of a wide range of physiological processes [73]. Moreover, the AQPs are able to transport metalloids such as boron and silicon, which are important for the growth of plants. By contrast, the transport of toxic substances such as arsenic and antimony leads to cell homeostasis [74]. It has been postulated that specific AQPs are also involved in the movement of the stomata. These pores perform the gas exchange process, which enables the CO_2_ uptake for photosynthesis at the expense of the water loss by transpiration [30]. In turn, it is also known that some AQPs transport glycerol. This compound acts as an osmolyte and its transport via the aquaporins may be part of the stress response [75]. The accumulation of osmolytes increases the suction power in cells, which provides protection against water leakage [76]. Furthermore, during the seed maturation and germination processes, the tissue water content changes and these fluctuations are ensured by the AQPs [75].

In general, the changes in the mRNA expression levels lead to changes in protein abundance, but sometimes this is not the case [59]. The downregulation of the gene expression could lead to decrease in protein abundance, which could further lead to decrease in the membrane permeability. In turn, the change in the membrane permeability could be obtain also by regulating the AQPs gating. Changes in the gene expression in response to environmental stimuli can be an important component of system homeostasis on the one hand, while the gene regulatory differences in response to the environment can be an important component of the adaptation to stress condition on the other [77]. The expression of AQPs can be altered by abiotic factors, including drought, cold, heat and salinity in plants. Lastly, regulating the AQPs expression and changes in membrane permeability could also have an effect on crop productivity. Examples of studies of the expression of the *AQP* genes under different abiotic stress in cereals are presented in Table 2. Among the AQPs, the members of the PIP and TIP subfamilies have been analysed most often in order to determine their expression patterns under abiotic stress in cereals. This is quite important when comparing the profile of the expression of *AQP* genes to pay attention to the developmental stage and stress treatment conditions that are used in different experiments. Each *AQP* isoform gene might have an individual pattern of expression due to playing different functions during plant growth; e.g., it was shown that OsPIP1;2 functions in CO_2_ diffusion rather than the modulation of water transport in rice [78]. The quantitative reverse transcription PCR (qRT-PCR) until now has been the most widely used approach for the discovery of the pattern of *AQP* gene expression in cereal species under abiotic stress (Table 2).

### 3.1. Drought and Heat Stress

Drought stress is one of the major abiotic stress and causes huge losses in crop yield. The periods of drought that are caused by climate change and their frequency and severity will continue to be a growing problem around the world [76,85]. The main function of aquaporins under drought stress is to precisely regulate the movement of water among cells and tissues [86]. An analysis of the *AQP* gene expression in the leaf and/or root tissues in barley (*Hordeum vulgare* L.), foxtail millet (*Setaria italica* L.), rice (*Oryza sativa* L.) and wheat (*Triticul aestivum* L.) revealed significant changes in their response to drought or dehydration stress, which had been induced by the application of polyethylene glycol-6000 (PEG) (Table 2) [61,66,75,80,81]. Applying 20% PEG is equivalent to osmotic potential levels of −1.09 MPa [87]. Under drought stress, a downregulation of the *AQP* genes in leaf tissue can lead to a decrease in the water permeability of the membrane to avoid water loss. This expression pattern has been shown in the leaves for some TIP family member genes in barley (*Hordeum vulgare* L.) after ten days of severe drought treatment (Table 2) [80]. Among them, HvTIP1;1, HvTIP1;2 and HvTIP2;3 have been proven experimentally by using their in vitro expression in yeast that are able to transport water [88]. Among cereal species, the same profile of expression has been observed for *OsTIP1;1* and *OsTIP2;2* in rice leaves after 10 h of treatment with 15% PEG-6000 [75]. 

The correlation between the root water uptake capacity (i.e., the root hydraulic conductivity Lpr) and *AQP* gene expression has been observed in many studies [89,90,91,92,93]. The first clue that aquaporins might play a major role in the overall root water uptake came from an experiment in which it was shown that Hg^2+^ decreased a plant’s root hydraulic conductivity, and Hg^2+^ is one of the inhibitors of aquaporins [94]. Aquaporin activity could affect root hydraulic properties under drought stress [82]. The contribution of aquaporins to the Lpr was generally high (up to 79% under well-watered conditions and 85% under drought stress) and was also differentially regulated under drought in rice [82]. It was suggested that the upregulation of root aquaporin expression could prevent inhibition of transpiration by increasing the root water flow [95,96]. The expression of *OsPIP2-4* and *OsPIP2-5* increased in the roots, while at the same time it decreased in the leaves after 24 h of treatment with 10% PEG in rice [81]. The same profile of expression- increase in the roots and decrease in the shoots was detected for *OsTIP1;1*, *OsTIP4;1* and *OsTIP4;2* after 10 h of treatment with 15% PEG in rice [75]. The identification the *AQP* genes whose expression was inversely regulated in the shoots and roots may be a result of different roles that are played by specific AQP in the various organs in plants.

Aquaporins also facilitate transport substrates other than water such as hydrogen peroxide (H_2_O_2_) or glycerol, which both might be produced under drought stress. Hydrogen peroxide is unstable and highly reactive molecules, which belongs to the reactive oxygen species (ROS) [97]. In turn, glycerol is an osmotic compound [76,98]. The expression of *HvTIP3;1* and *HvTIP4;1* genes increased under drought stress in barley leaves [80]. This profile of expression was associated with the involvement of HvTIP3;1 in moving hydrogen peroxide. The transport of this substrate by this AQP was predicated in barley by bioinformatic analysis [64]. In barley, using the bioinformatic analysis the substrate other than water that can be transported by HvTIP4;1 has not been identified [64]. However, in rice it was demonstrated that its orthologue, OsTIP4;1, has a glycerol transport activity using the *Xenopus* oocyte expression system [75]. The increased expression of *HvTIP4;1* under drought stress might also be connected with an increased transport of glycerol. In rice, a nine-fold increase in the expression of *OsTIP4;1* was detected only in the shoots after 8 h of PEG-6000 treatment compared to the control conditions [75]. Other investigated durations of treatment of 2, 4, 6 and 10 h did not cause such big changes in the amount of the transcript [75].

Drought stress often occurs simultaneously with high temperatures in field conditions. Because of global climate change, temperature stress is becoming a major area of concern for researchers around the world [99]. Foxtail millet (*Setaria italica* L.) is a model plant for studying biofuels, stress tolerance and C_4_ photosynthetic traits [66]. The profile of expression of the aquaporin genes during heat stress in this species was investigated in the roots. After 24 h of treatment at 45 °C, all of the analysed genes, which are members of PIP, TIP, NIP and SIP subfamilies, had an increase in the abundance of these transcripts (Table 2) [66]. This might suggest that an increased uptake of water by the roots is needed to cool the temperature of leaves by increasing transpiration.

### 3.2. Cold Stress

Plants are exposed to a wide range of environmental conditions, and in addition to heat, cold stress is also a factor that shapes the physiology of cereals [99]. Low soil temperatures inhibit water uptake by roots [100]. A decrease in water permeability across the root (i.e., a decrease in the root hydraulic conductivity, Lpr) is expected to affect the plant water status, stomatal conductance, photosynthesis, growth and, lastly, productivity [94,101,102,103,104]. In rice, a decrease in the Lpr under cold stress was linked to the role of aquaporins [105]. A 96-h treatment at 4 °C was enough to cause a decrease in the expression of eight *PIP* and two *TIP* genes in rice (Table 2) [63]. Interestingly, the differences in the profiles of the *AQP* genes (nine *PIPs* and three *TIPs*) expression was also detected in rice when whole plants were chilled compared to when only the roots of the plants were chilled at 10 °C for five days. When only the roots were chilled, there was an increased expression of *OsPIP1;3*, *OsPIP2;4* and *OsPIP2;5* compared to the control conditions (Table 2) [79]. The authors observed that a low room temperature treatment (LRT) for a prolonged length of time induced a gradual increase the root osmotic hydraulic conductivity Lpr(os), which suggests that LRT-treated roots acclimate to low temperatures by acquiring a water uptake mechanism that is not present at the normal growth temperature of 25 °C [79]. This might suggest that a cold acclimation process for the root water uptake functions in rice and that is possibly regulated via aquaporins [79]. Furthermore, the low temperature causes changes in the amount of AQP transcripts not only in roots but also in leaves. Recently, it was shown that temperature of growth was a significant factor influencing the level of accumulation of HvPIP1 transcript and protein in barley leaves. The level of accumulation of HvPIP1 transcript decreased at 5 °C (compared to 20 °C) but was higher at 27 °C than at 20 °C in the analysed genotypes [106].

### 3.3. Salinity

Soil salination and a high salt accumulation affect the growth, development, metabolism and yield of plants [107]. A wide range of physiological and biochemical alterations in plants is induced by salinity, which causes a lower water potential in the soil solution and ionic disequilibrium, as well as a higher accumulation of reactive oxygen species (ROS) [107]. This is why the salt stress tolerance trait has multi-component nature [108]. Among cereals, barley is highly tolerant for salinity, while wheat and maize display a high sensitivity [34]. AQPs could play a role in the mechanism by which plants cope with saline concentration because it leads to changes in the *AQP* gene expression profiles, which has been shown in many plant species including cereals (Table 2). In barley (*Hordeum vulgare* L.), foxtail millet (*Setaria italica* L.) and rice (*Oryza sativa* L.), different concentrations of NaCl from 150 till 250 mM were used for the salt treatment, as well as different durations of the treatment (Table 2). During the early research on barley, the opposite profile of the expression of the *HvPIP2;1* gene was detected in the roots and shoots after 48 h of treatment with 200 mM NaCl, which decreased and increased compared to the control conditions, respectively (Table 2) [109]. The HvPIP2;1 protein was confirmed to be localised in the plasma membrane. The functional expression of this gene in *Xenopus* oocyte indicated that HvPIP2;1 has a water transport activity [83]. Other research on both barley and rice also showed that after 24 h of NaCl treatment, there was a decrease in the expression of many members of the PIP subfamily in the roots (Table 2) [81,84]. Severe salinity stress (200 mM NaCl) also significantly reduced the root hydraulic conductivity (Lpr) in rice [84]. However, under relatively mild salt stress (100 mM NaCl), there was only a moderate decrease in the Lpr with no significant difference in the *HvPIP* mRNA levels [84]. The biological significance of such a regulation may increase the prevention of an excessive water loss from cells during the early stages (the osmotic stress phase) of salinity stress, and the Lpr decrease might represent the mode of change in barley plants from the rapid growth stage, which requires a high water absorption to the protected stage, which requires less water uptake [84]. Conversely, under salinity stress some *TIP* genes were detected whose expression increased compared to the control conditions such as *OsTIP1;1* and *OsTIP1;2* (Table 2) [75]. OsTIP1;2 had a water and glycerol transport activity [58]. The upregulation of some of the *OsTIPs* genes might be connected with water transport from the vacuole to the cytoplasm to perform an osmotic adjustment [75].

### 3.4. Comparison between Genotypes, Which Are Characterised as Tolerant or Resistant to Specific Abiotic Stress

An extremely interesting aspect of an expression study of *AQP* genes under abiotic stress conditions is the comparison between the cultivars, which are characterised by the opposite phenotype in terms of their tolerance to specific abiotic stresses. This kind of research could lead to identifying the important players among AQPs in stress tolerance. The genes that would be identified in such a way could serve as potential candidates for further downstream characterisation studies in order to understand the molecular mechanisms that underlie abiotic stress tolerance. Two contrasting cultivars of the foxtail millet (*Setaria italica* L.), which is a naturally abiotic stress-tolerant crop, were investigated in terms of their abiotic stress tolerance. The study showed that *SiPIP3;1* and *SiSIP1;1* were differentially expressed in both of the cultivars in response to salinity, heat and dehydration stresses [66]. In the stress-tolerant cultivar, the expression of these genes in roots were higher compared to the resistant ones. Their role in providing salinity and dehydration stress tolerance was also validated via the heterologous overexpression of these genes in the *S. cerevisiae* system in which the overexpression lines were found to be tolerant to abiotic stresses [66]. In turn, in rice, the expression of the aquaporin *RWC3* gene (PIP1) was investigated in upland and lowland rice. Upland rice is traditionally regarded as having drought avoidance, which is one of the mechanisms of drought resistance [110]. The expression of *RWC3* mRNA was higher in the upland rice in the early response (up to four hours) to a 20% polyethylene glycol (PEG) 6000 treatment in the roots, whereas there were no significant changes in its expression in the lowland rice. The protein levels increased in the upland rice and decreased in the lowland rice after a ten-h water deficit. An analysis with the *Xenopus* oocyte expression system showed that RWC3 has a water transport activity. The authors suggested that a high level of *RWC3* improves water uptake under a water deficit and therefore maintains the plant water balance [111]. Another interesting example of an *AQP* gene expression study in two inbred lines that contrast in their water use efficiency, root hydraulic conductivity (Lpr) and AQP contribution to the Lpr was performed on pearl millet (*Pennisetum glaucum* (L) R. Br.). This is a key cereal for food security in arid and semi-arid regions of the world, which is heat and drought adapted [112]. Increased water use efficiency (WUE) can alleviate drought stress [67]. The line with a lower WUE had a significantly higher AQP contribution to the Lpr and also had a higher *PgPIP1-3* and *PgPIP1-4* gene expression compared to the line with a higher WUE that was grown in hydroponic conditions. The authors suggested that a downregulation of the *PgPIP* genes might induce inhibition of transpiration and water savings. However, an investigation of the expression patterns of the *AQP* genes under abiotic stress conditions could be a very important subject for further research. Additionally, treating roots with a common AQP inhibitor (azide) suggested that AQP contributed up to 84% of the root hydraulic conductivity (Lpr) in pearl millet [67]. Furthermore, in the same cereal, four recombinant inbred lines that vary in their transpiration rate (Tr) response to vapour pressure deficit (VPD) conditions were selected for an expression study of the *AQP* genes [113]. Some of the water-saving traits were the capacity of certain genotypes to restrict plant transpiration when the evaporative requisition was high [114]. The differences in the transpiration response phenotype were accompanied by diverse *AQP* gene abundances between the VPD-sensitive and VPD-insensitive genotypes. The expression pattern of the *PgAQP* genes indicated that the VPD-insensitive genotypes had a slightly higher expression of AQPs, including *PgPIP1;1* and *PgPIP2;6* in the root tissue, than in the VPD-sensitive genotypes under low VPD conditions (1.2 kPa), when there was no high evaporative demand from the atmosphere [113]. The last example is from wheat (*Triticum aestivum* L.) for which the profiles of the *AQP* genes were investigated using accessions with contrasting responses to drought according to the yield tolerance index (YTI). In the leaves, as stress intensity increased, some of the *AQP* genes of the PIP1 and PIP2 subfamilies such as *TaPIP1-1*, *TaPIP1-5* or *TaPIP2-24* were clearly upregulated in the tolerant genotype. In the case of the TIP family, *TaTIP2-4*, *TaTIP3-4* and *TaTIP4-1* also increased their expression levels during severe stress. On the other hand, in the roots, the tolerant genotype had a lower level of expression compared to the susceptible ones for the *TaPIP1-1*, *TaPIP1-5*, *TaAQP7*, *TaPIP2-1C1*, *TaTIP1-2*, *TaTIP3-4* and *TaTIP4-1* genes under moderate stress [61].

## 4. Overexpression and Mutant Lines in the Cereal Aquaporin-Encoding Genes

The *AQP* genes are a potential target for crop improvement, which is primarily connected with their functions in regulating the water balance in plants. Only members of the plasma membrane intrinsic proteins (PIP) and nodulin-26-like proteins (NIP) family genes from cereals have been reported to be involved in increasing the tolerance to abiotic stress in several plant species using genetic modification technology (Table 3). With the most common plants, the goal of the research was to manipulate the *AQP* gene expression to increase the tolerance to either drought or salt stress or both. Genetically manipulating the cereal *AQP* genes that belong to the PIP1 group such as *RWC3*, *OsPIP1;1*, *OsPIP1;2* and *TaAQP8* was performed and transgenic plants were generated in rice, Arabidopsis and tobacco [78,81,111,115,116]. In turn, in the PIP2 group, *PsPIP2;2*, *TaAQP7*, *TdPIP2;1* and *ZmPIP2;5*, were investigated and transgenic plants were created in Arabidopsis, tobacco, wheat and maize [81,117,118,119]. An increased drought or salt stress tolerance of transgenic plants was reported based on an increase in some parameters after growth in stress conditions such as the germination rate, root growth, biomass production, root osmotic hydraulic conductivity (Lp), relative water potential (RWC) in the leaves, transpiration, leaf elongation rate, retained low Na^+^ and high K^+^ concentrations in the shoots, a lower H_2_O_2_ accumulation and less membrane damage because the antioxidant system was improved (Table 3). Maintaining transpiration is important in plants, especially during drought stress, because it facilitates the dissipation of excess heat. This effect was obtained by the overexpression of *Oryza sativa RWC3* in rice, and the relative cumulative transpiration after ten hours of treatment with 20% PEG 6000 was higher compared to the wild type plants (WT) [111]. Interestingly, the transgenic plants with an overexpression of *Hordeum vulgare HvPIP2;5* in Arabidopsis were able to survive and recover after a three-week period of drought [120]. Manipulating the expression of the *AQP* genes does not always lead to the intended results. There is one example in which the overexpression of the *Hordeum vulgare HvPIP2;1* gene resulted in an increased sensitivity to abiotic stress. Transgenic rice plants with an overexpression of this gene were more sensitive to 100 mM NaCl [109]. In turn, the overexpression of *OsPIP1;2* in rice improved not only growth, but importantly also grain yield by facilitating leaf CO_2_ diffusion, which increases both the net CO_2_ assimilation rate and sucrose transport [78]. Not only were plants with an overexpression of some *AQP* genes created, but those with the knockout of *AQP* gene were also investigated in order to improve the plant response to abiotic stress. Rice is a major source of toxic metalloid arsenic in food. The arsenic accumulation in rice grains leads to food contamination. The knockout of either the *Oryza sativa OsNIP1;1* or *OsNP3;3* gene had little effect on arsenite uptake or translocation. However, the overexpression of the genes did decrease the root-to-shoot translocation of arsenite and the shoot arsenic concentration by disrupting the radial transport of arsenite in the roots. When they were grown in arsenic-contaminated paddy soils, the transgenic plants contained significantly lower arsenic concentrations in their rice grains [121].

## 5. Concluding Remarks

According to the Intergovernmental Panel on Climate Change report, the global mean temperature increased by 0.8 °C in the 20th century and is predicted to increase by 3–5 °C in the 21st century [122]. This means that extreme daily temperatures and heat waves might be a growing problem for sustainable agriculture in the future. Not only increases in temperature but also predicted increases in soil salinisation and levels of CO_2_ in the atmosphere will effect cereal growth and yield [123,124].

Water is a fundamental compound for living all organisms. It is believed that aquaporins may be important players in the plant water relations at the cell, tissue, organ and whole plant levels. AQPs may fine-tune the water transport through its transcriptional regulation and establish a suitable water balance under stress conditions. Therefore, these proteins may play a crucial role in the stress response as well as in stress tolerance and might also serve as a target sequence for genetic modifications.

Different studies have associated the changes in the *AQPs* profiles of expression with an adaptive mechanism that limits the effects of different abiotic stresses. A functional analysis of these candidate genes or proteins will be necessary to draw adequate conclusions. Taken together, AQPs probably play complex and diversified roles in the response of plants to abiotic stresses that are dependent on specific isoforms and the type and degree of stress treatment. In order to improve the tolerance of cereals to abiotic stress conditions, a better understanding of the AQP family members and their functions in plant physiology is needed.

## Figures and Tables

**Table 1:** Number of aquaporin (AQP) isoforms in the genome of cereal species and its area harvested.

Species	Area Harvested (ha) [60]	Genome Size/Ploidy x/ No. of Chromosomes	AQP	PIP	TIP	NIP	SIP	References
Bread wheat (*Triticum aestivum* L.)	215,901,958	~17,000 Mb 2n = 6x = 42 AABBDD hexaploid	113 (65-A, 42-B, 36-D)	51	29	29	4	[61]
Maize (*Zea mays* L.)	197,204,250	2400 Mb 2n = 2x = 20	41	12	18	8	3	[62]
Rice Japonica (*Oryza sativa*)	162,055,938	500 Mb 2n = 2x = 24	33	11	10	10	2	[63]
Barley (*Hordeum vulgare* L.)	51,149,869	~5300 Mb 2n = 2x = 14	39	18	11	8	2	[64]
Sorghum (*Sorghum bicolor* (L.) *Moench*)	40,074,667	~730 Mb 2n = 2x = 20	37	13	11	11	2	[65]
Foxtail millet (*Setaria italica*)	31,653,878 ^	~490 Mb 2n = 2x = 18	39	12	11	13	3	[66]
Pearl millet (*Pennisetum glaucum* (L.) R Br.)	31,653,878 ^	~1790 Mb 2n = 2x = 14	33	10	9	11	3	[67]

^ both harvested millet areas together.

**Table 2:** Study of the expression the *AQP* genes in cereal species under abiotic stress.

Abiotic Stress	Cereal Species	Stage of Growth	Treatment	Method	Analysed *AQP* Genes	Effect on the Expression Level/Tissue		References
						Increased	Decreased	
Cold	Rice (*Oryza sativa* L. spp. *japonica*)	16-week-old seedlings	4 °C treatment, 96 h	RT-PCR **	8 PIPs 2 TIPs		Roots: *OsPIP1;1*, *OsPIP1;2*, *OsPIP2;1*, *OsPIP2;2*, *OsPIP2;3*, *OsPIP2;4*, *OsPIP2;5*, *OsPIP2;6*, *OsTIP1;1* *OsTIP2;2*	[63]
	Rice (*Oryza sativa* L. spp. *japonica*)	16–20-day-old seedlings	Root chilling: culture solution set in a water bath maintained at °C; whole-plant chilling: plants were placed in a 10 °C set growth chamber, 5 days ^	qRT-PCR *	9 PIPs 3 TIPs	Roots chilled: *OsPIP1;3* *OsPIP2;4 OsPIP2;5*	Roots chilled: *OsPIP2;6* *OsTIP1;1* *OsTIP2;2* Whole plant chillled: *all analysed*	[79]
Drought/dehydratation	Barley (*Hordeum vulgare* L.)	24-day-old seedlings	ten days of drought in soil under a volumetric water content of 1.5%	qRT-PCR *	8 TIPs	Leaves: *HvTIP3;1 HvTIP4;1*	Leaves: *HvTIP1;1* *HvTIP1;2* *HvTIP2;1* *HvTIP2;2* *HvTIP2;3*	[80]
	Foxtail millet (*Setaria italica* L.), tolerant cultivar	21-day-old seedlings	Cultivated with 20% PEG 6000, 24 h ^	qRT-PCR *	2 PIPs 1 TIP 1 NIP 1 SIP	Roots: *SiPIP3;1* *SiSIP1;1* *SiTIP2;2*	Roots: *SiPIP1;2* *SiNIP1;2*	[66]
	Rice (*Oryza sativa* L. spp. *japonica*)	two-week-old seedlings	Cultivated with 10% PEG 6000, 24 h ^	qRT-PCR *	10 PIPs	Roots: *OsPIP1-1* *OsPIP1-2* *OsPIP1-3* *OsPIP2-4* *OsPIP2-5* *OsPIP2-7*	Leaves: *OsPIP2-1* *OsPIP2-4* *OsPIP2-5* *OsPIP2-6*	[81]
	Rice (*Oryza sativa* L. spp. *japonica*)	four-week-old seedlings	Cultivated hydroponically with 15% PEG 6000, 10 h ^	qRT-PCR *	7 TIPs	Root: *OsTIP1;1* *OsTIP1;2* *OsTIP4;1* *OsTIP4;2*	Shoot: *OsTIP1;1* *OsTIP2;2* *OsTIP4;1* *OsTIP4;2*	[75]
	Rice (*Oryza sativa* L.), 6 rice varieties	29-day-old seedlings	Drought in soil	qRT-PCR * in Azucena variety (japonica group) ^	10 PIPs		Roots: All analysed	[82]
	Wheat (*Triticum aestivum* L.)	one-week-old seedlings	cultivated with 20% PEG-6000, 6 h ^	Microarray data obtained from WheatExp database	113 AQPs	17 *PIPs* 12 *TIPs* Examples *: *TaTIP1-2* *TaTIP1-4* *TaTIP2-3* *TaTIP2-4* *TaTIP3-3* *TaTIP3-4* *TaTIP3-6* *TaTIP4-1* *TaTIP4-4* *TaTIP4-5*	16 *PIPs* 3 *SIPs* Examples *: *TaNIP1-3 TaNIP1-4* *TaNIP1-6 TaNIP2-1C1* *TaTIP2-2 TaTIP4-1C1* *TaPIP2-1* *TaPIP2-3* *TaPIP2-4C1* *TaPIP2-6* *TaPIP2-10* *TaPIP2-12* *TaPIP2-14* *TaPIP2-19*	[61]
	Wheat (*Triticum aestivum* L.), drought-tolerant genotype compared to the control conditions	Anthesis stage (Z61) —grain filling	Drought stress—without irrigation	qRT-PCR *	8 PIPs 4 TIPs 2 NIPs	Leaves: *TaPIP1-1 TaPIP1-5 TaPIP2-24* Roots: *TaPIP1-1* *TaPIP1-5* *TaPIP2-2C1* *TaTIP1-2* *TaTIP2-4* *TaTIP3-4* *TaNIP4-3* *TaLEA*	Leaves: *TaNIP4-2* Roots: TaPIP1-6 TaPIP2-7 TaPIP2-24 TaNIP4-1	[61]
Heat	Foxtail millet (*Setaria italica* L.), tolerant cultivar	21-day-old seedlings	45 °C treatment, 24 h	qRT-PCR *	2 PIPs 1 TIP 1 NIP 1 SIP	Roots: *SiPIP1;2* *SiPIP3;1* *SiSIP1;1* *SiTIP2;2* *SiNIP1;2*		[66]
Salinity	Barley (*Hordeum vulgare* L.)	four-day-old seedlings	cultivated with 200 mM NaCl, 48 h	RT-PCR **	3 PIPs	Shoots: *HvPIP2;1*	Roots: *HvPIP2;1*	[83]
	Barley (*Hordeum vulgare* L.)	four-day-old seedlings	cultivated with 200 mM NaCl, 24 h	qRT-PCR *	10 PIPs		Roots: *HvPIP1;2*, *HvPIP1;3*, *HvPIP1;4*, *HvPIP2;1*, *HvPIP2;2 HvPIP2;3*	[84]
	Foxtail millet (*Setaria italica* L.), tolerant cultivar	21-day-old seedlings	cultivated with 200 mM NaCl, 24 h	qRT-PCR *	2 PIPs 1 TIP 1 NIP 1 SIP	Roots: *SiPIP1;2* *SiPIP3;1* *SiSIP1;1* *SiTIP2;2*	Roots: *SiNIP1;2*	[66]
	Rice (*Oryza sativa*)	two-week-old seedlings	cultivated with 250 mM NaCl, 24 h ^	qRT-PCR *	10 PIPs	Leaves: *OsPIP1-2* Roots: *OsPIP1-1* *OsPIP1-2* *OsPIP2-4*	Leaves: *OsPIP1-1* *OsPIP2-1* *OsPIP2-3* *OsPIP2-4* *OsPIP2-6* Roots: *OsPIP1-3* *OsPIP2-2* *OsPIP2-3* *OsPIP2-5*	[81]
	Rice (*Oryza sativa* L. spp. *japonica*)	four-week-old seedlings	cultivated hydroponically with 150 mM NaCl, 10 h ^	qRT-PCR *	7 TIPs	Roots: *OsTIP1;1* *OsTIP1;2* *OsTIP4;1* *OsTIP4;2*	Shoots: *OsTIP1;2* *OsTIP2;2* *OsTIP4;2* *OsTIP4;3* Roots: *OsTIP2;2* *OsTIP4;3*	[75]

* Quantitative real time PCR (qRT-PCR); ** semi-quantitative RT-PCR, end-point technique; ^ the time of treatment that was selected; treatment with PEG 6000 was used to mimic the drought stress.

**Table 3:** Genetic manipulation of the *AQP* genes of cereals and its effect on the plant phenotype.

Species	*AQP* Gene	Method/Expression in Species	Promoter	Improved Tolerance to Abiotic Stress/or Other Traits	Phenotype	References
*Hordeum vulgare* L.	*HvPIP2;1*	OX *Oryza sativa*	CaMV35S	No	In control conditions, there was an increased radial hydraulic conductivity of roots (Lpr) of up to 140% and the mass ratio of the shoot to root of up to 150%. Under salt stress of 100 mM NaCl, reduction in growth was greater than in non-transgenic plants	[109]
	*HvPIP2;5*	OX *Arabidopsis thaliana*	CaMV35S	Yes	Better stress tolerance during germination and root growth under high salt and high osmotic stresses. Able to survive and recover after a three-week drought	[120]
*Oryza sativa* L.	*RWC3* (PIP1 group)	OX *Oryza sativa,* lowland	SWPA2	Yes	Better water status under a water deficit. Increased root osmotic hydraulic conductivity (Lp), leaf water potential and relative cumulative transpiration at the end of ten-h treatment with 20% PEG 6000	[111]
	*OsPIP1;1*	OX *Oryza sativa*	CaMV35S	Yes	Increased seed yield, salt resistance, root hydraulic conductivity and seed germination rate	[115]
	*OsPIP1;1* *OsPIP2;2*	OX *Arabidopsis thaliana*	CaMV35S	Yes	Improved tolerance to salt (100 mM of NaCl) and drought (200 mM of mannitol), but not to salt treatment at a higher concentration (150 mM of NaCl)	[81]
	*OSPIP1;2*	OX *Oryza sativa*	CaMV35S	Yes	Improved growth and grain yield by facilitating leaf CO_2_ diffusion, which increases both the net CO_2_ assimilation rate and sucrose transport.	[78]
	*OsNIP1;1,* *OsNIP3;3*	OX KO *Oryza sativa*	OsLsi1	Yes	Knockout of either gene had little effect on arsenite uptake or translocation. Overexpression did not affect arsenite uptake but decreased the root-to-shoot translocation of arsenite and shoot arsenic concentration. When grown in arsenic-contaminated paddy soils, there was a significantly lower arsenic concentration in the rice grains	[121]
*Triticum aestivum/* *turgidum* L.	*TaAQP7* (PIP2 group)	OX *Nicotiana tabacum*	CaMV35S	Yes	Increased drought tolerance. Lower levels of malondialdehyde (MDA) and H_2_O_2_ and less ion leakage (IL), but a higher relative water content (RWC) and superoxide dismutase (SOD) and catalase (CAT) activities	[117]
	*TaAQP8* (PIP1 group)	OX *Nicotiana tabacum*	CaMV35S	Yes	Increased root elongation compared to the controls under salt stress. Retaining a high K^+^/Na^+^ ratio and Ca^2+^ content, but also lowering the H_2_O_2_ accumulation and membrane damage by improving the antioxidant system	[116]
	*TdPIP2;1*	OX *Triticum durum*	PrCaMV35S	Yes	Improved germination rates and biomass production and retained low Na^+^ and high K^+^ concentrations in the shoots under high salt and osmotic stress conditions. A long-term study under greenhouse conditions on salt or drought stress produced good quality grains	[118]
*Zea mays* L.	*ZmPIP2;5*	OX KO *Zea mays*	CaMV35S	Yes	Whole-root conductivity decreased in the KO lines; no difference was observed in the OX plants. At the leaf level, the hydraulic conductance was higher in the PIP2;5 OE lines whereas there was no difference in the pip2;5 KO lines. Leaf elongation rate was faster in the PIP2;5 OE plants after mild drought stress	[119]

OX—overexpression, KO—knockout, PEG—polyethylene glycol, CaMV35S—the cauliflower mosaic virus p35S promoter.

## Data Availability

The study did not report any data.
